# An evolutionary differential game for regulating the role of monoclonal antibodies in treating signalling pathways in oesophageal cancer

**DOI:** 10.1098/rsos.240347

**Published:** 2024-07-31

**Authors:** Mesfer Alajmi, Souvik Roy

**Affiliations:** ^1^ Department of Mathematics, The University of Texas at Arlington, Arlington, TX 76019-0407, USA

**Keywords:** evolutionary game theory, Nash equilibrium, Stackelberg equilibrium, evolutionary resistance, relaxation scheme, nonlinear conjugate gradient

## Abstract

This work presents a new framework for a competitive evolutionary game between monoclonal antibodies and signalling pathways in oesophageal cancer. The framework is based on a novel dynamical model that takes into account the dynamic progression of signalling pathways, resistance mechanisms and monoclonal antibody therapies. This game involves a scenario in which signalling pathways and monoclonal antibodies are the players competing against each other, where monoclonal antibodies use Brentuximab and Pembrolizumab dosages as strategies to counter the evolutionary resistance strategy implemented by the signalling pathways. Their interactions are described by the dynamical model, which serves as the game’s playground. The analysis and computation of two game-theoretic strategies, Stackelberg and Nash equilibria, are conducted within this framework to ascertain the most favourable outcome for the patient. By comparing Stackelberg equilibria with Nash equilibria, numerical experiments show that the Stackelberg equilibria are superior for treating signalling pathways and are critical for the success of monoclonal antibodies in improving oesophageal cancer patient outcomes.

## 1. Introduction

Oesophageal cancer (OC) ranks as the sixth most prevalent cause of cancer-related mortality on a global scale [1]. The signalling pathways within OC, encompassing monovalent ligands like epidermal growth factor (EGF) receptor and the complex receptor formed by the combination of the epidermal growth factor receptor (EGFR) and EGF, are of utmost importance in regulating cellular viability, proliferation and differentiation [2]. Genetic mutations frequently cause dysregulation of cancer cell signalling pathways, which in turn causes cancer cells to become resistant to treatment [3]. Several potential points of inhibition can be found along the signalling pathways, they have the potential to specifically target receptors located on the cellular membrane [4]. Monoclonal antibodies (mAbs), also known as immunotherapy, are the most effective inhibitors for OC signalling pathways in preventing resistance development. Monoclonal antibodies demonstrate a remarkable level of specificity, meaning that each antibody binds exclusively to a single target [5]. Pembrolizumab is a monoclonal antibody that specifically targets the checkpoint protein PD-1 on the surface of T cells, a type of immune cell. The mechanism of action involves the inhibition of the interaction between the checkpoint protein PD-L1, located on the surface of tumour cells, and its associated signalling pathways. This mechanism enables T to attack and eliminate cancerous cells [6]. Moreover, Pembrolizumab also has the bonus of stimulating immune checkpoints, which play a crucial role in modulating the immune response. Until they are required, T cells are normally ‘off’, or inactive, owing to immune checkpoints. As a result, the T cells are suppressed in their attempts to harm the healthy tissues. The goal of developing and using mAbs has been to increase the efficiency of therapeutic agent delivery to tumour sites. Brentuximab is a monoclonal antibody that has been linked to chemotherapy agents. It works by blocking signalling pathways and transporting a chemotherapeutic agent, preventing cancer cells from spreading and multiplying [7]. Understanding the Darwinian mechanisms that drive the evolutionary dynamics of signalling pathways is proving to be a promising avenue for developing new approaches to treating this disease [8–12]. Using evolutionary game theory (EGT) to model the evolution of treatment resistance in signalling pathways is crucial for reaching this objective.

The study of biological interactions between players is a fruitful application of the theoretical framework of EGT [13]. In an evolutionary game, each participant represents a distinct species or population. EGT examines the dynamics between species that use varying tactics and/or characteristics. These organisms do not need to act rationally, by contrast to classical game theory, because their strategies are inherited rather than deliberately selected. These strategies possess the potential to enhance an organism’s fitness, which is a measure of its ability to survive and proliferate. Consequently, individuals employing these strategies are more inclined to eventually attain population dominance [14]. In an evolutionary game, a player’s success or failure depends on how well they can strategically respond to their opponent’s actions. Differential games are problems within the field of EGT that focus on modelling and studying conflicts that arise in a dynamic framework [15]. The process of organismal evolution over time can be more accurately described by dynamical models, which are typically modelled using a system of differential equations.

Various deterministic models have been employed to simulate the dynamics of signalling pathways in diverse cancer types. The mathematical model proposed by Itano *et al*. [16] uses ordinary differential equations (ODEs) to investigate the dimerization mechanism underlying the development of Gefitinib resistance in lung cancer. Bianconi *et al*. [17] employ an ODE-based model to examine the correlation between the expressions of EGFR and IGF1R proteins in non-small-cell lung cancer. Cross-talk between the oestrogen receptor and the EGFR is described using a mathematical model introduced in [18]. A computational model presented in [19] simulates biochemical and metabolic interactions observed in melanoma cancer between the PI3K/AKT and MAPK pathways. In [20], the authors investigate how AKT pathways contribute to therapy resistance in receptor tyrosine kinase (RTK) signalling in colon cancer. In a very recent work [21], the authors propose an optimal control framework to determine the best treatment strategies for controlling aberrant RTK signalling pathways in EC patients. However, all the aforementioned models do not adequately describe the evolutionary dynamics of treatment-resistant signalling pathways in OC.

Some recent clinical trials have shown that treatment protocols based on evolutionary principles lead to better clinical outcomes. In [22,23], it has been shown that bipolar androgen therapy anticipates the development of resistance to androgen deprivation therapy (ADT) in advanced prostate cancer. By strategically administering androgen, it aims to restore sensitivity to ADT. In another clinical trial, it was demonstrated that even though small-cell lung cancers might develop resistance to immunotherapy, at the same time, they exhibit increased response to cytotoxicity [24]. These clinical trials demonstrate the feasibility of an evolutionary game-theoretic framework in improving clinical outcomes of cancer patients.

Motivated by the aforementioned evolutionary frameworks in other cancers and the fact that one of the primary reasons for failure of clinical trials in OC is attributed to drug resistance by cancer [25], our work focuses on an evolutionary game-theoretic framework modelling interactions of treatments and resistance in OC to improve clinical outcomes. In this context, the interaction between mAbs and signalling pathways in the context of treating OC can be analogized to a differential game [26]. The signalling pathways exhibit evolutionary modifications and exhibit adaptive responses to the treatment administered by mAbs, using various mechanisms to evade the intended therapeutic effects of the medication. The game’s design confers a notable advantage upon the monoclonal antibodies. The signalling pathways exhibit a limited capacity to anticipate or adapt to therapeutic interventions that have not yet been administered. However, mAbs exhibit their capacity to predict the subsequent advancement of the signalling pathways. As a result, the game demonstrates a notable imbalance in power [27]. Therefore, mAbs initiate the first action by delivering treatment, while the signalling pathways subsequently respond by developing resistance to it. In other words, the signalling pathways’ ability to implement adaptive strategies is inactive until the administration of a particular treatment. Within this particular context, mAbs can be regarded as assuming a leadership role, while the signalling pathways can be seen as taking on a follower position. Therefore, the treatment of the signalling pathways can be classified as a Stackelberg game.

Nevertheless, the existing treatment protocols for signalling pathways, such as the continuous administration of the maximum tolerated dose (MTD), fail to effectively exploit the advantage or disparity in the game [11]. In the context of signalling pathways therapies, repeated utilization of a consistent approach significantly increases the probability of the signalling pathways developing resistance towards the treatment. In this scenario, mAbs cannot see the signalling pathways move because the game is played simultaneously by all players. As a result, mAbs hands over the reins of leadership to the signalling pathways when they show signs of making progress. In the given context, the concept of Nash equilibrium or Nash solution emerges, wherein both the mAbs and signalling pathways cannot independently alter their strategies in a manner that would result in a favourable outcome for either party [15]. Currently, there is no established and all-encompassing framework for an evolutionary differential game in the context of signalling pathways in OC. The goal of this research is to examine a class of game-theoretic formulations that can be used to determine the optimal course of treatment for a patient with OC.

This paper is organized as follows: §2 introduces an ODE model that aims to provide a comprehensive understanding of the evolutionary process of signalling pathway resistance. In §3, we show the theoretical formulation of an evolutionary differential game, which includes the analysis of both Stackelberg and Nash equilibria. Section 4 is devoted to the analysis of the existence of Nash and Stackelberg equilibria. The numerical schemes that are proposed to resolve Nash and Stackelberg equilibria are shown in §5. In §6, numerical simulations are presented to support our analytical results. Finally, in §7, conclusions are presented.

## 2. An ordinary differential equation model for signalling pathways in oesophageal cancer

The presented model elucidates the mechanisms by which immunotherapies modulate signalling pathways. It explains how T cells can be directed and effectively administer chemotherapy to destroy signalling pathways through mAbs strategies. The model also predicts an expansion of the signalling pathways owing to their evolutionary resistance towards immunotherapies. Our model is constructed based on the law of mass action, with additional insights from Reed *et al*. [28]. We first define

—
$\hat{L}(\hat{t})$ —the density of EGF ligand (no./volume)
—
$\hat{C}(\hat{t})$ —the density of EGF:EGFR complex (no./volume)
—
$\hat{T}(\hat{t})$ —the concentration of T cells per litre of blood (cells l^−1^)
—
$\hat{M}(\hat{t})$ —the concentration of chemotherapy per litre of blood (mg l^−1^)
—
${\hat{u}}_{b}(\hat{t})$ —the dosage of Brentuximab per litre of blood (mg l^−1^)
—
${\hat{u}}_{p}(\hat{t})$ —the dosage of Pembrolizumab per litre of blood (mg l^−1^)
—
${\hat{u}}_{c}(\hat{t})$ —the evolutionary resistance strategy of the signalling pathways (no./volume).

The governing equations of a mathematical model are given as follows:


(2.1) $$\begin{array}{l} \frac{\text{d}\hat{L}}{\text{d}\hat{t}}=\hat{\gamma }\hat{L}+\frac{1}{2}{\hat{a}}_{1}\hat{\alpha }\hat{L}\hat{C}+{\hat{a}}_{2}\hat{C}-{\hat{a}}_{3}\hat{\alpha }{\hat{u}}_{p}\hat{L}\hat{T},\;\hat{L}(0)={\hat{L}}_{0}\\ \frac{\text{d}\hat{C}}{\text{d}\hat{t}}={\hat{b}}_{1}(\hat{\alpha }-\frac{1}{2}\hat{C})\hat{L}-\frac{1}{2}{\hat{b}}_{2}({\hat{H}}_{0}+\hat{C})\hat{C}+\frac{1}{2}{\hat{b}}_{3}({\hat{H}}_{0}-\hat{C})-{\hat{b}}_{4}\frac{{\hat{u}}_{b}}{\hat{k}+\hat{b}{\hat{u}}_{c}}\hat{C},\;\hat{C}(0)={\hat{C}}_{0}\\ \frac{\text{d}\hat{T}}{\text{d}\hat{t}}=\hat{\omega }-{\hat{d}}_{1}(\hat{L}+\hat{C})\hat{T}+{\hat{d}}_{2}{\hat{u}}_{p}\hat{T},\;\;\hat{T}(0)={\hat{T}}_{0}\\ \frac{\text{d}\hat{M}}{\text{d}\hat{t}}=-{\hat{d}}_{3}\hat{M}+{\hat{d}}_{4}{\hat{u}}_{b},\;\hat{M}(0)={\hat{M}}_{0}, \end{array}$$

where $H_{0}$ and $R_{0}$ are the initial conditions for the epidermal growth factor receptor HER2 and EGFR, respectively [29], and $\alpha =R_{0}-\frac{1}{2}H_{0}$ . The following are descriptions of the terms used in (2.1):

—
$\hat{\gamma }\hat{L}$ —the exponential EGF ligand growth,
—
$\frac{1}{2}{\hat{a}}_{1}\hat{\alpha }\hat{L}\hat{C}$ —the rate of change of $\hat{L}$ is made up of a gain rate proportional to $\hat{L}\hat{C}$ ,
—
${\hat{a}}_{2}\hat{C}$ —the rate of change of $\hat{L}$ is made up of a gain rate proportional to $\hat{C}$ ,
—
$-{\hat{a}}_{3}\hat{\alpha }{\hat{u}}_{p}\hat{L}\hat{T}$ —Pembrolizumab stimulates $\hat{T}$ cells, causing the death of EGF ligand $\hat{L}$ ,
—
${\hat{b}}_{1}(\hat{\alpha }-\frac{1}{2}\hat{C})\hat{L}$ —the gain rate proportional to $\hat{R}\hat{L}$ makes up the rate of change of $\hat{C}$ ,
—
$-\frac{1}{2}{\hat{b}}_{2}({\hat{H}}_{0}+\hat{C})\hat{C}$ —the rate of change of $\hat{C}$ is made up of a loss rate proportional to $\hat{H}\hat{C}$ ,
—
$\frac{1}{2}{\hat{b}}_{3}({\hat{H}}_{0}-\hat{C})$ —the gain rate proportional to EGF : EGFR : HER2 complex makes up the rate of change of $\hat{C}$ ,
—
$-{\hat{b}}_{4}\frac{{\hat{u}}_{b}}{\hat{k}+\hat{b}{\hat{u}}_{c}}\hat{C}$ —Brentuximab transports chemotherapy, preventing complex $\hat{C}$ formation in the presence of evolutionary resistance,
—
$-{\hat{d}}_{1}\left(\hat{L}+\hat{C}\right)\hat{T}$ —death of cells owing to the signalling pathways interactions,
—
${\hat{d}}_{2}{\hat{u}}_{p}\hat{T}$ —the amount of Pembrolizumab injection needed for activating T cells,
—
$-\hat{d_{\text{3}}}\hat{M}$ —the excretion and elimination of chemotherapy toxicity,
—
${\hat{d}}_{4}{\hat{u}}_{b}$ —the amount of Brentuximab injected.

The other parameters are described as follows:

—
$\hat{\gamma }$ —the growth rate of monovalent ligands (EGF) (day^−1^),
—
${\hat{a}}_{1}$ —the gain rate proportional to $\hat{L}\hat{C}$ (cell),
—
${\hat{a}}_{2}$ — the gain rate proportional to $\hat{C}$ complex (cell),
—
${\hat{a}}_{3}$ - the rate of $\hat{L}$ death caused by T cells (l^2^ cells^-1^ mg^-1^),
—
${\hat{b}}_{1}$ —the gain rate proportional to $\hat{R}\hat{L}$ (cell),
—
${\hat{b}}_{2}$ —the loss rate proportional to $\hat{H}\hat{C}$ (cell),
—
${\hat{b}}_{3}$ —the gain rate proportional to EGF : EGFR : HER2 complex (cell),
—
${\hat{b}}_{4}$ —the rate of $\hat{C}$ death caused by specific drug (l mg^-1^),
—
$\hat{k}$ —the rate of the natural resistance that may be present before drug exposure,
—
$\hat{b}$ —the rate of the benefit the cell gains by reducing sensitivity to the drug,
—
$\hat{\omega }$ —the rate of circulating T cells (cell),
—
${\hat{d}}_{1}$ —the rate of T cells death owing to signalling pathways (cell^−1^ day^−1^),
—
${\hat{d}}_{2}$ —the rate of the amount of Pembrolizumab injected (mg l^−1^),
—
${\hat{d}}_{3}$ —the rate of chemotherapy drug decay (day^−1^),
—
${\hat{d}}_{4}$ —the rate of the amount of Brentuximab injected (mg l^−1^).

It is essential to use the following non-dimensionalized variables to non-dimensionalize the above ODE system to improve the numerical algorithms’ stability,


(2.2) $$L=q_{1}\hat{L},C=q_{2}\hat{C},T=q_{3}\hat{T},M=q_{4}\hat{M}\;t=q_{5}\hat{t},u_{p}=q_{6}{\hat{u}}_{p},u_{c}=q_{7}{\hat{u}}_{c},u_{b}=q_{8}{\hat{u}}_{b}$$

and the corresponding parameters are


(2.3) $$\begin{array}{c} \gamma =\frac{\hat{\gamma }}{q_{5}},\;a_{1}=\frac{{\hat{a}}_{1}}{q_{2}},\;\alpha =\frac{\hat{\alpha }}{q_{5}},\;a_{2}=\frac{{\hat{a}}_{2}q_{1}}{q_{2}q_{5}},\;a_{3}=\frac{{\hat{a}}_{3}}{q_{3}q_{6}},\;H_{0}=\frac{{\hat{H}}_{0}}{q_{5}}\\ b_{1}=\frac{q_{2}{\hat{b}}_{1}}{q_{1}q_{5}},\;b_{2}=\frac{{\hat{b}}_{2}}{q_{2}},\;b_{3}={\hat{b}}_{3}q_{2},\;b_{4}=\frac{{\hat{b}}_{4}}{q_{5}q_{8}},\;k=\hat{k}q_{7},\;b=\frac{\hat{b}}{q_{7}}\\ \omega =\frac{\hat{\omega }q_{3}}{q_{5}},\;d_{1}=\frac{{\hat{d}}_{1}}{q_{5}q_{3}},\;d_{2}=\frac{{\hat{d}}_{2}}{q_{5}q_{6}},\;d_{3}=\frac{{\hat{d}}_{3}}{q_{5}},\;d_{4}=\frac{{\hat{d}}_{4}q_{4}}{q_{5}q_{8}}. \end{array}$$

In this context, the scaling weights $q_{i},\;i=1,\ldots ,8$ serve the purpose of non-dimensionalizing the parameters and model variables, as well as ensuring that they possess comparable ranges. The system has been transformed into a non-dimensionalized form, which is expressed as


(2.4) $$\begin{array}{l} \frac{dL}{dt}=\gamma L+\frac{1}{2}a_{1}\alpha LC+a_{2}C-a_{3}\alpha u_{p}LT,\;L\left(0\right)=L_{0}\\ \frac{dC}{dt}=b_{1}\left(\alpha -\frac{1}{2}C\right)L-\frac{1}{2}b_{2}\left(H_{0}+C\right)C+\frac{1}{2}b_{3}\left(H_{0}-C\right)-b_{4}\frac{u_{b}}{k+bu_{c}}C,\;C\left(0\right)=C_{0}\\ \frac{dT}{dt}=\omega -d_{1}\left(L+C\right)T+d_{2}u_{p}T,\;T\left(0\right)=T_{0}\\ \frac{dM}{dt}=-d_{3}M+d_{4}u_{b},\;M\left(0\right)=M_{0}. \end{array}$$

Let $x={\left(L,C,T,M\right)}^{T}$ and $u={\left(u_{p},u_{b},u_{c}\right)}^{T}$ . Then, the ODE system in (2.4) can be expressed as


(2.5) $$\begin{array}{c} \dot{x}=f\left(x,u\right),\\ x\left(0\right)=x_{0}. \end{array}$$

In §3, we use this model to create two evolutionary differential games involving mAbs and signalling pathways.

## 3. An evolutionary differential game

A differential game is said to be complete in the context of EGT if all players are fully aware of one another’s strategy spaces and cost functionals [30]. In this framework, we consider a situation in which the two players, each driven by their self-interest, have no desire to work together. We build a complete evolutionary differential game in which the mAbs and the signalling pathways are the game’s players, denoted by A and S , respectively. In (3.1), $\Omega_{1}$ and $\Omega_{2}$ represent the spaces of admissible strategies for A , while $\Omega_{3}$ represents the space of admissible strategies for S .


(3.1) $$\begin{array}{c} \Omega_{1}=\left\{u_{p}\in L^{2}\left(\left[0,T_{f}\right],R\right):0\leq u_{p}\left(t\right)\leq D_{1},\;\forall t\in \left[0,T_{f}\right]\right\}\\ \Omega_{2}=\left\{u_{b}\in L^{2}\left(\left[0,T_{f}\right],R\right):0\leq u_{b}\left(t\right)\leq D_{2},\;\forall t\in \left[0,T_{f}\right]\right\}\\ \Omega_{3}=\left\{u_{c}\in L^{2}\left(\left[0,T_{f}\right],R\right):0\leq u_{c}\left(t\right)\leq D_{3},\;\forall t\in \left[0,T_{f}\right]\right\}. \end{array}$$

We observe that $\Omega_{1},\Omega_{2}$ and $\Omega_{3}$ are closed and convex. In (3.1), $D_{1}$ and $D_{2}$ represent the MTDs of Pembrolizumab and Brentuximab, respectively, which can be administered to achieve optimal outcomes in eradicating the signalling pathways. If the MTDs for a given patient are exceeded, there is a risk to the patient’s health [31]. When cancer cells can divide and pass on their genetic mutations, a strong selective pressure is generated, pushing the cells to become resistant to treatment. The development of resistance would be stymied if this factor were not present [32]. Consequently, we set the upper limit of evolutionary resistance to be $D_{3}$ . The model presented in (2.5) is a playground setting in which A and S employ their strategies (3.1) to surpass one another. The period of the game’s development is represented by the interval $\left[0,T_{f}\right]$ .

The primary objective of mAbs is the eradication of cancerous cells. To achieve this, they employ strategies that effectively limit the number of potential signalling pathways. Furthermore, it effectively mitigates the adverse consequences induced by T cells. Therefore, A endeavours to minimize its own objective functional, namely


(3.2) $$\underset{\Omega_{1}\times \Omega_{2}}{\text{arg min}}\;J_{A}\left(x,u\right):=\int_{0}^{T_{f}}\left(\left(\tau -1\right){\left[G-\left(L+C\right)\right]}^{2}-\tau {\left(Z-u_{b}\right)}^{2}-ru_{p}T\right)dt+\int_{0}^{T_{f}}\left(\mu u_{b}^{2}+\eta u_{p}^{2}\right)dt.$$

Five terms are stated in (3.2). In the first and second terms, maximum tumour burden G is shown to be correlated with signalling pathways abundance, and maximum chemotherapy dose Z is shown to be correlated with Brentuximab, where τ is the chemotherapy toxicity rate. The third term represents how Pembrolizumab regulates T cells, where r represents the Pembrolizumab’s toxicity. Regularization priors for Brentuximab and Pembrolizumab costs are given by the fourth and fifth terms in $J_{A}$ , with μ,η≥0 denoting the corresponding regularization weight. If the disease exhibits stabilization or reduction in size without complete eradication, the administration of treatment will persist as long as it remains well-tolerated and the dissemination of signalling pathways is effectively contained. In the event of cancer progression, the administration of treatment will be discontinued. Therefore, it can be inferred that G,L or C possess greater values compared with Z , $u_{b}$ , $u_{p}$ or T , where τ≥1 , and r is a positive parameter. Hence, $J_{A}$ is bounded below by 0 . If such an outcome fails to materialize, the game will reach its conclusion.

In relation to the primary objective of S in this game, it is to evade death by developing resistance to the treatment being used. So S aims to minimize his own objective functional, namely


(3.3) $$\underset{\Omega_{3}}{\text{arg min}}\;J_{S}\left(x,u\right):=\int_{0}^{T_{f}}\left[\gamma L+C+\frac{u_{b}}{k+bu_{c}}\right]dt+\nu \int_{0}^{T_{f}}u_{c}^{2}\;dt.$$

There are four different possible terms in (3.3). The first three terms, which together determine how to fit a solution of $J_{\text{S}}$ and incorporate the strategies into the evolutionary process, present the fitness of signalling pathways [33]. The fourth term in $J_{\text{S}}$ is the regularization term that represents the cost of the signalling pathways resistance, and ν≥0 is the associated regularization weight. Note that $J_{\text{S}}$ is bounded below by 0 .

Given the aforementioned preparation, a non-cooperative infinite evolutionary differential game involving mAbs and the signalling pathways can be formulated within the framework of the calculus of variations [15] as follows:


(3.4) $$\begin{array}{l} \underset{\Omega_{1}\times \Omega_{2}}{\text{arg min}}\;J_{A}\left(x,u\right)\\ \underset{\Omega_{3}}{\text{arg min}}\;J_{S}\left(x,u\right)\\ \text{s}\text{.t.}\;\;\dot{x}=f\left(x,u\right),x\left(0\right)=x_{0}. \end{array}$$

**Figure 1.**
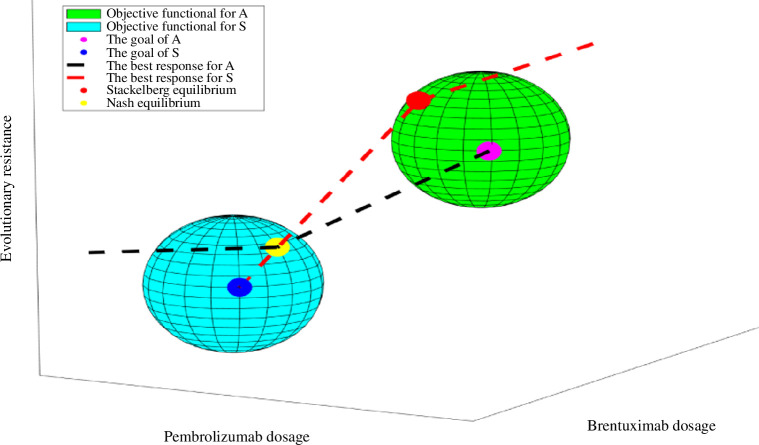
A visual representation interprets potential outcomes arising from the interplay of signall properties of the cost funcing pathways and mAbs.


Solving (3.2) will result in the most effective responses of A to counter emerging resistance from S . Moreover, solving of (3.3) would result in the optimal responses of S in relation to the administered treatments. We consider the following best-response maps (see figure 1).

(3.5) $$\begin{array}{c} R_{A}\left(u_{c}\right)=\left\{\left(u_{p},u_{b}\right):\left(u_{p},u_{b}\right)=\underset{\Omega_{1}\times \Omega_{2}}{\text{arg min}}\;J_{A}\right\}\\ R_{S}\left(u_{p},u_{b}\right)=\left\{u_{c}:u_{c}=\underset{\Omega_{3}}{\text{arg min}}\;J_{S}\right\}. \end{array}$$

Accurately assessing the players’ knowledge levels at any given moment is imperative for a comprehensive understanding of the game. The availability of information significantly impacts a player’s decision-making process. Hence, the results of the game may exhibit significant variability contingent upon how the treatment is administered. In the following subsections, we will analyse the potential outcomes of the game (AS).

### 3.1. Stackelberg equilibrium

In the game (AS), the administration of therapy by mAbs serves as the initial action. Subsequently, the signalling pathways respond by generating countermeasures through the process of evolving their resistance. Although resistance-related molecular machinery may have been present before treatment commenced, it is possible that it did not undergo selective pressure in the form of a resistance mechanism until treatment was initiated [34]. Consequently, the therapy of signalling pathways can be conceptualized as a strategic interaction between a leader and a follower. The investigation of leader–follower dynamics was first conducted by von Stackelberg [13], revealing notable benefits for the leader. The mAbs’ initial intervention as a leader, leveraging their ability to anticipate the subsequent responses of the signalling pathways, offers a pivotal chance to attain more advantageous outcomes through the strategic guidance and limitation of resistance mechanisms employed by these signalling pathways. A Stackelberg equilibrium (SE) necessitates that player A strategically determines its optimal outcome by considering the best-response curve of player S (see figure 1).

More precisely, $\left(x^{*},u_{p}^{*},u_{b}^{*},u_{c}^{*}\right)\in {\left(H^{1}\left(0,T_{f}\right)\right)}^{4}\times \Omega_{1}\times \Omega_{2}\times R_{S}$ is a SE of the game (AS) if the following conditions hold:

-
$u_{c}^{*}=R_{S}\left(u_{p}^{*},u_{b}^{*}\right)$

- For any $\left(u_{p},u_{b}\right)\in \Omega_{1}\times \Omega_{2}$ and every best-response $u_{c}\in R_{S}$ ,

(3.6) $$J_{A}\left(x^{*},u_{p}^{*},u_{b}^{*},u_{c}^{*}\right)\leq J_{A}\left(x,u_{p},u_{b},u_{c}\right).$$

### 3.2. Nash equilibrium

If the monoclonal antibodies cannot take advantage of the asymmetry in the game (AS) by taking the initiative, mAbs will lose the ability to do both predictive and directive work. As a result, the mAbs employ a consistent approach by repetitively administering drugs at maximum doses, even though the signalling pathways continually develop effective adaptive reactions [26]. Moreover, by adopting a treatment approach that is solely based on modifying the treatment following the progression of the signalling pathways, mAbs effectively surrender control to these pathways, consequently heightening the probability of treatment ineffectiveness. The signalling pathways and mAbs employ strategies that demonstrate progression along their respective best-response curves as they engage in an iterative process of moves and countermoves. The given situation results in the formation of a Nash equilibrium (NE), which is identified by the point where the two curves intersect (see figure 1). Neither the signalling pathways nor the mAbs can make strategic changes that would benefit them individually in the context of NE.

From a mathematical perspective, $\left(\bar{x},{\bar{u}}_{p},{\bar{u}}_{b},{\bar{u}}_{c}\right)\in {\left(H^{1}\left(0,T_{f}\right)\right)}^{4}\times \Omega_{1}\times \Omega_{2}\times \Omega_{3}$ is a NE of the game (3.4) if the following conditions hold:


(3.7) $$\begin{array}{ll} \left(\bar{x},{\bar{u}}_{p},{\bar{u}}_{b},{\bar{u}}_{c}\right)\;\; & =\underset{\Omega_{1}\times \Omega_{2}}{\text{arg min}}\;J_{A}\left(x,u_{p},u_{b},{\bar{u}}_{c}\right)\\ & =\underset{\Omega_{3}}{\text{arg min}}\;J_{S}\left(x,{\bar{u}}_{p},{\bar{u}}_{b},u_{c}\right), \end{array}$$

where $\left({\bar{u}}_{p},{\bar{u}}_{b}\right)=R_{A}\left({\bar{u}}_{c}\right)$ ; and ${\bar{u}}_{c}=R_{S}\left({\bar{u}}_{p},{\bar{u}}_{b}\right)$ . Let $\Omega =\Omega_{1}\times \Omega_{2}\times \Omega_{3}$ , and define the controls-to-state map


(3.8) $$\Lambda :\Omega \to {\left(H^{1}\left(0,T_{f}\right)\right)}^{4},u\to x,$$

and consider the following reduced functionals:


(3.9) $${\hat{J}}_{A}\left(u_{p},u_{b},u_{c}\right):={\hat{J}}_{A}\left(\Lambda \left(u_{p},u_{b},u_{c}\right),u_{p},u_{b},u_{c}\right)\;{\hat{J}}_{S}\left(u_{p},u_{b},u_{c}\right):={\hat{J}}_{C}\left(\Lambda \left(u_{p},u_{b},u_{c}\right),u_{p},u_{b},u_{c}\right).$$

Then, $\left({\bar{u}}_{p},{\bar{u}}_{b},{\bar{u}}_{c}\right)$ is a NE for the game (AS) if the following holds:


(3.10) $$\begin{array}{l} {\hat{J}}_{A}\left({\bar{u}}_{p},{\bar{u}}_{b},{\bar{u}}_{c}\right)\leq {\hat{J}}_{A}\left(u_{p},u_{b},{\bar{u}}_{c}\right),\;\left(u_{p},u_{b}\right)\in \Omega_{1}\times \Omega_{2}\\ {\hat{J}}_{S}\left({\bar{u}}_{p},{\bar{u}}_{b},{\bar{u}}_{c}\right)\leq {\hat{J}}_{S}\left({\bar{u}}_{p},{\bar{u}}_{b},u_{c}\right),\;u_{c}\in \Omega_{3}. \end{array}$$

When it comes to achieving NE, the treatment of single pathways presents a significant challenge for mAbs. However, success can be achieved through the use of the right strategy. Using the game’s inherent asymmetry, mAbs could improve patient outcomes and reduce side effects without resorting to unsafely high doses of Pembrolizumab or Brentuximab.

## 4. Theory of the evolutionary differential game

We provide a theoretical analysis of the NE and SE for the differential game (AS). In [35–38], there are analogous findings for other NE and SE differential games and optimal control problems. First, we prove that the ODE system (2.5) has positive solutions.

**Lemma 4.1.** *The solution* x *of (* 2.5 *) is non-negative if the initial condition* $x_{0}$ *is non-negative for all* $t\in [0,T_{f}]$ .

*Proof.* We can write (2.5) as follows:


(4.1) $$\frac{dx}{dt}=R\left(x,u\right)-M\left(x,u\right)x,$$

where


$$R\left(x,u\right)=\left[\begin{array}{c} \gamma L+\frac{1}{2}a_{1}\alpha LC+a_{2}C\\ b_{1}\left(\alpha -\frac{1}{2}C\right)L+\frac{1}{2}b_{3}\left(H_{0}-C\right)\\ \omega +d_{2}u_{p}T\\ d_{4}u_{b} \end{array}\right],\;M\left(x,u\right)x=\left[\begin{array}{c} a_{3}\alpha u_{p}T\\ \frac{1}{2}b_{2}\left(H_{0}+C\right)+b_{4}\frac{u_{b}}{k+bu_{c}}\\ d_{1}\left(L+C\right)\\ d_{3} \end{array}\right]{\left[\begin{array}{cccc} L & C & T & M \end{array}\right]}^{T}.$$

If x,u≥0 , we obtain R,M≥0 , componentwise. By multiplying both sides of (4.1) by the integrating factor vector I=exp(∫M(x,u) dt) , we obtain that


$$I\frac{dx}{dt}+\text{exp}\left(\int M\left(x,u\right)\;dt\right)M\left(x,u\right)x=IR\left(x,u\right).$$

This gives us


(4.2) $$\frac{d\left(Ix\right)}{dt}=IR\left(x,u\right)\geq 0.$$

Since, $x_{0}\geq 0,$ we have ${Ix}_{0}\geq 0$ . Thus, (4.2) gives us that Ix(t)≥0 for all $t\in [0,T_{f}]$ . Since, I>0 , we have that x(t)≥0 for all $t\in [0,T_{f}]$ .∎

We next show some stability estimates for the solution of (2.5).

**Lemma 4.2.** *A solution* x *of (* 2.5 *) satisfies the following stability estimate:*


(4.3) $$\begin{array}{l} L\left(t\right)\leq \text{exp}\left(\int_{0}^{T_{f}}\gamma +\frac{1}{2}a_{1}\alpha W\;\text{d}t\right)\left[\int_{0}^{T_{f}}a_{2}W\;\text{exp}\;\left(\int_{0}^{T_{f}}-\gamma -\frac{1}{2}a_{1}\alpha W\;\text{d}s\right)\;dt+L_{0}\right],\\ C\left(t\right)\leq W,\\ T\left(t\right)\leq T_{0}\;\text{exp}\;\left(d_{2}D_{1}t\right)+\frac{\omega }{d_{2}D_{1}}\left[\text{exp}\;\left(d_{2}D_{1}t\right)-1\right],\\ M\left(t\right)\leq d_{4}D_{2}t+M_{0}, \end{array}$$

*where* $W=C_{0}\;exp\;\left(\int_{0}^{T_{f}}b_{1}\alpha L\left(s\right)+b_{3}H_{0}\;ds\right).$

*Proof*. From (2.5), we note the following:


$$\begin{array}{l} \frac{dL}{dt}\leq \gamma L+\frac{1}{2}a_{1}\alpha LC+a_{2}C,\\ \frac{dC}{dt}\leq b_{1}\alpha L+b_{3}H_{0},\\ \frac{dT}{dt}\leq \omega +d_{2}u_{p}T\leq \omega +d_{2}D_{1}T,\\ \frac{dM}{dt}\leq d_{4}u_{b}\leq d_{4}D_{2}. \end{array}$$

A simple application of Gronwall’s inequality gives the desired result.∎

Lemma 4.2 gives us that a solution x of (2.5) is bounded. We now state and prove the existence and uniqueness of solutions of (2.5).

**Theorem 4.1.** *Given* u∈Ω *, there exists a unique solution* x *of (2.5) in* ${\left(H^{1}(0,T_{f})\right)}^{4}$ *.*

Proof. Since u∈Ω , u is bounded. From lemma 4.2, we have that x is bounded. Let $f\left(x\right)={\left(f_{1}\left(x\right),f_{2}\left(x\right),f_{3}\left(x\right),f_{4}\left(x\right)\right)}^{T}$ , then we also compute the following gradients:


$$\begin{array}{l} \nabla_{x}f_{1}(x)=\left(\gamma +\frac{1}{2}a_{1}\alpha C-a_{3}\alpha u_{p}T,\frac{1}{2}a_{1}\alpha L+a_{2},-a_{3}\alpha u_{p}L,0\right),\\ \nabla_{x}f_{2}(x)=\left(b_{1}(\alpha -\frac{1}{2}C),-\frac{1}{2}b_{1}L-\frac{1}{2}b_{2}H_{0}-b_{2}C-\frac{1}{2}b_{3}-b_{4}\frac{u_{b}}{k+bu_{c}},0,0\right),\\ \nabla_{x}f_{3}(x)=\left(-d_{1}T,-d_{1}T,-d_{1}(L+C)+d_{2}u_{p},0\right),\\ \nabla_{x}f_{4}(x)=\left(0,0,0,-d_{3}\right). \end{array}$$

We have $\|\nabla_{x}f_{i}\left(x\right){\|}_{\infty },\;i=1,2,3$ is bounded. Thus f is Lipschitz. Therefore the following conditions are satisfied by f :

-
f is continuous with respect to x .
-
f is measurable with respect to t .
-
f is bounded.
- The derivative of f with respect to x is also bounded.

Thus, f satisfies the Caratheodory’s conditions, and so there is a unique solution $x\in {\left(H^{1}\left(0,T_{f}\right)\right)}^{4}$ of (2.5). ∎

We now have some properties of the cost functionals $J_{A},J_{S}$ given in (3.2) and (3.3). Similar arguments can be found in [39,40].

**Proposition 1.** *The objective functionals* $J_{A},J_{S}$ *, given in (3.2) and (3.3), are sequentially weakly lower semi-continuous (w.l.s.c.), bounded from below, coercive on* $\Omega_{1},\Omega_{2},\Omega_{3}$ *, and Fréchet differentiable.*

*Proof*. We will address the properties of $J_{S}$ and similar arguments will also hold for $J_{A}$ . The following are the steps to proving the properties of $J_{S}$ :

1. $J_{S}$ is bounded below by 0 and is coercive since $lim_{||u_{c}||\to \infty }\;J_{S}\left(u_{c}\right)=\infty$ . Also, $\Omega_{3}$ is weakly sequentially compact in $L^{2}$ . To see this, let $\left\{u_{c}^{n}\right\}\in \Omega_{3}$ such that $||u_{c}^{n}|{|}_{L^{2}}\leq D_{3}$ for all n , so it is bounded. Thus, there is a subsequence $\left\{u_{c}^{n_{k}}\right\}$ such that $u_{c}^{n_{k}}⇀u_{c}\in \Omega_{3}$ . Also, we have that $||u_{c}|{|}_{L^{2}}\leq lim\;inf||u_{c}^{n}|{|}_{L^{2}}\leq D_{3}$ . Thus, $u_{c}\in \Omega_{3}$ . Next we consider the sets


$$U_{\alpha }=\left\{u_{c}\in \Omega_{3}:J_{s}\left(u_{c}\right)\leq \alpha \right\}.$$

Since $J_{S}$ is continuous, the sets $U_{\alpha }$ are closed for all α∈ℝ . Thus, $U_{\alpha }$ is a closed subset of a weakly sequentially compact space $\Omega_{3}$ and is weakly sequentially closed for all α∈ℝ. This implies that $J_{S}$ is weakly sequentially lower semi-continuous.

2. Let $u_{c}^{1}$ ,and $u_{c}^{2}$ be functions in $\Omega_{3}$ and $\sigma \in [0,T_{f}]$ . By Minkowski inequality, we have


$$\|\sigma u_{c}^{1}+(1-\sigma )u_{c}^{2}\|\leq \sigma \|u_{c}^{1}\|+(1-\sigma )\|u_{c}^{2}\|\leq \sigma D_{3}+(1-\sigma )D_{3}=D_{3}.$$

Hence, $\Omega_{3}$ is convex.

3. Let $\left\{u_{c}^{n}\right\}\in \Omega_{3}$ such that $u_{c}^{n}\to u_{c}\in L^{2}([0,T_{f}],\mathbb{R})$ . Let ϵ>0 , then, by the reverse Minkowski inequality, $\left|\left\|u_{c}^{n}\|-\|u_{c}\right\|\right|<\|u_{c}^{n}-u_{c}\|<\epsilon$ . Thus, $\left\|u_{c}^{n}\|\to \|u_{c}\right\|$ , and since $\left\{u_{c}^{n}\right\}\in \Omega_{3}$ , $\|u_{c}^{n}\|\leq D_{3}$ , and so, $\|u_{c}\|\leq D_{3}$ . Hence, $\Omega_{3}$ is closed.

4. The Frechet differential of the operator $J_{S}$ at $u_{c}$ is the bounded linear operator


$$A\left(h\right)=\int_{0}^{T}\frac{u_{b}}{bu_{c}+\left(k+bh\right)}+2\nu u_{c}h-\frac{u_{b}}{bu_{c}+k}dt.$$

To show that A is the Frechet differential of $J_{S}$ at $u_{c}$ , we have the following:


$$\begin{array}{l} \underset{||h||\to 0}{\lim}\frac{|J_{S}(u_{c}+h)-J_{S}(u_{c})-A(h)|}{||h|{|}_{L^{2}}}\\ =\underset{||h||\to 0}{\lim}\frac{1}{||h|{|}_{L^{2}}}\left|\int_{0}^{T}\left[\frac{u_{b}}{k+b(u_{c}+h)}+\nu {\left(u_{c}+h\right)}^{2}-\frac{u_{b}}{k+bu_{c}}-\nu u_{c}^{2}-\frac{u_{b}}{bu_{c}+(k+bh)}-2\nu u_{c}h+\frac{u_{b}}{bu_{c}+k}\right]\;dt\right|\\ =\underset{||h||\to 0}{\lim}\frac{1}{||h|{|}_{L^{2}}}\left|\int_{0}^{T}\nu h^{2}\;dt\right|\\ \leq \underset{||h||\to 0}{\lim}\frac{1}{||h|{|}_{L^{2}}}\int_{0}^{T}\nu |h^{2}|\;dt\\ \leq \underset{||h||\to 0}{\lim}\frac{||h|{|}_{L^{2}}^{2}}{||h|{|}_{L^{2}}}\int_{0}^{T}\nu \;dt\\ =0. \end{array}$$

Also, A(h) is a linear operator by the linearity of the integral. To see that A(h) is bounded, we use Hölder inequality,


$$\begin{array}{ll} ||A(h)|{|}_{L^{2}}^{2} & ={\left|\int_{0}^{T}\frac{u_{b}}{bu_{c}+(k+bh)}+2\nu u_{c}h-\frac{u_{b}}{bu_{c}+k}\;\text{d}t\right|}^{2}\\ & ={\left|\int_{0}^{T}\frac{-bhu_{b}}{\left(bu_{c}+k)[bu_{c}+(k+bh)\right]}+2\nu u_{c}h\;\text{d}t\right|}^{2}\\ & ={\left|\int_{0}^{T}h\left[\frac{-bu_{b}}{\left(bu_{c}+k)[bu_{c}+(k+bh)\right]}+2\nu u_{c}\right]\;\text{d}t\right|}^{2}\\ & \leq \int_{0}^{T}|h{|}^{2}dt\int_{0}^{T}{\left|\frac{-bu_{b}}{\left(bu_{c}+k)[bu_{c}+(k+bh)\right]}+2\nu u_{c}\right|}^{2}\;\text{d}t\\ & ={\left\|h\right\|}_{L^{2}}^{2}{\left\|\frac{-bu_{b}}{\left(bu_{c}+k)[bu_{c}+(k+bh)\right]}+2\nu u_{c}\right\|}_{L^{2}}^{2}. \end{array}$$

Thus,


$$\begin{array}{ll} \|A\left(h\right){\|}_{L^{2}} & \leq \|h{\|}_{L^{2}}\|\frac{-bu_{b}}{\left(bu_{c}+k\right)\left[bu_{c}+\left(k+bh\right)\right]}+2\nu u_{c}{\|}_{L^{2}}\\ & \leq \|h{\|}_{L^{2}}\left[\|\frac{bu_{b}}{bu_{c}+k}{\|}_{L^{2}}+\|2\nu u_{c}{\|}_{L^{2}}\right]\\ & \leq \|h{\|}_{L^{2}}\left[\frac{b}{k}D_{2}+2\nu D_{3}\right]. \end{array}$$

Therefore, A is the Frechet differential of the operator $J_{S}$ at $u_{c}$ . In a similar way, one can show that $J_{A}$ satisfies the properties in proposition 1. ∎

Owing to the objective functionals in (3.2) and (3.3) being non-convex, Nash’s theorem cannot be used to prove the existence of a NE. The following theorem establishes that a NE of our game (AS) is a solution to a specified control problem. We then demonstrate that an optimal solution exists for this control problem.

The composite cost functional is defined as follows:


(4.4) $$\hat{J}\left(u_{p},u_{b},u_{c}\right)={\hat{J}}_{A}\left(u_{p},u_{b},u_{c}\right)+{\hat{J}}_{S}\left(u_{p},u_{b},u_{c}\right),$$

and we consider the optimal control problem


(4.5) $$\text{arg min}\;\hat{J}\left(u_{p},u_{b},u_{c}\right),\;\;\left(u_{p},u_{b},u_{c}\right)\in \Omega .$$

**Theorem 4.2.** *If there is a minimizer* $\left({\bar{u}}_{p},{\bar{u}}_{b},{\bar{u}}_{c}\right)$ *of (4.5), then* $\left({\bar{u}}_{p},{\bar{u}}_{b},{\bar{u}}_{c}\right)$ *is a NE of the game (AS).*

*Proof.* We have that $\hat{J}({\bar{u}}_{p},{\bar{u}}_{b},{\bar{u}}_{c})\leq \hat{J}(u_{p},u_{b},u_{c})$ for all $(u_{p},u_{b},u_{c})\in \Omega$ . Thus,


$${\hat{J}}_{A}\left({\bar{u}}_{p},{\bar{u}}_{b},{\bar{u}}_{c}\right)+{\hat{J}}_{S}\left({\bar{u}}_{p},{\bar{u}}_{b},{\bar{u}}_{c}\right)\leq {\hat{J}}_{A}\left(u_{p},u_{b},u_{c}\right)+{\hat{J}}_{S}\left(u_{p},u_{b},u_{c}\right).$$

Let $\left(u_{p},u_{b},u_{c})=({\bar{u}}_{p},{\bar{u}}_{b},{\bar{u}}_{c}\right)$ in $J_{S}$ and $u_{c}={\bar{u}}_{c}$ in $J_{A}$ . Then, we obtain


$${\hat{J}}_{A}\left({\bar{u}}_{p},{\bar{u}}_{b},{\bar{u}}_{c}\right)\leq {\hat{J}}_{A}\left(u_{p},u_{b},{\bar{u}}_{c}\right),\;\;\left(u_{p},u_{b}\right)\in \Omega_{1}\Omega_{2}.$$

Likewise, we can get


$${\hat{J}}_{S}\left({\bar{u}}_{p},{\bar{u}}_{b},{\bar{u}}_{c}\right)\leq {\hat{J}}_{S}\left({\bar{u}}_{p},{\bar{u}}_{b},u_{c}\right),\;\;u_{c}\in \Omega_{2}.$$

Thus, the requirements of definition (3.10) have been satisfied.

With this preparation, we are now ready to state and prove the main results of the existence of Nash and Stackelberg equilibria. The proofs use similar arguments given in [35–38]. ∎

**Theorem 4.3.** *Let* $\hat{J}$ *be given as in (4.4). Then, there exists a pair* $\left(\bar{x},\bar{u}\right)\in {\left(H^{1}\left(0,T_{f}\right)\right)}^{4}\times \Omega$ *such that* $\bar{x}$ *is a solution of (2.5), and* $\bar{u}$ *minimize* $\hat{J}$ *in* Ω .

*Proof*. We define a map $\Lambda :\Omega \to {\left(H^{1}\left(0,T_{f}\right)\right)}^{4}$ by Λ(u)=x . By theorem 4.1 and lemma 4.2, we have that Λ is weakly sequential continuous. Since $\hat{J}$ is bounded from below, there exist minimizing sequences $\left(x^{k},u^{k}\right)\in {\left(H^{1}\left(0,T_{f}\right)\right)}^{4}\times \Omega$ such that $\Lambda \left(u^{k}\right)=x^{k}$ , where $x^{k}$ is the corresponding sequence of states. Since $\hat{J}$ is coercive, and Λ is bounded, we have that $\left(x^{k},u^{k}\right)$ is bounded. By using Eberlein–Šmulian theorem, there are weakly convergent subsequences $\left(u^{k_{l}}\right)$ and $\left(x^{k_{l}}\right)$ such that


$$\left(u^{k_{l}}\right)⇀\left(\bar{u}\right)\in \Omega \;\text{and}\;,\left(x^{k_{l}}\right)⇀\bar{x}\in {\left(H^{1}\left(0,T_{f}\right)\right)}^{4}.$$

Since the compact embedding $H^{1}(0,T_{f})\subset \subset C(0,T_{f})$ , the Rellich–Kondrachov theorem implies that


$$\left(x^{k_{l}}\right)\to \bar{x}\in L^{2}\left(\left[0,T_{f}\right]\right).$$

Now, we need to verify that $\bar{u}$ , and $\bar{x}$ satisfy $\Lambda \left(\bar{u}\right)=\bar{x}$ . Let $\phi \in H^{1}(0,T_{f})$ be a test function that is compactly supported. Then, since Λ is bounded and the variable state x is bounded by lemma 4.2, we can apply the dominated convergence theorem.


$$\int_{0}^{T_{\text{f}}}\Lambda \left(\bar{u}\right)\;\phi dt=\underset{k\to \infty }{\text{lim}}\int_{0}^{T_{\text{f}}}\Lambda \left(u^{k_{\text{l}}}\right)\;\phi dt=\underset{k\to \infty }{\text{lim}}\int_{0}^{T_{\text{f}}}x^{k_{\text{l}}}\phi =\int_{0}^{T_{\text{f}}}\bar{x}\;\phi dt,$$

thus


$$\int_{0}^{T_{\text{f}}}\left(\Lambda \left(\bar{u}\right)-\bar{x}\right)\;\phi dt=0\;\;\forall \phi \in H^{1}\left(0,T_{\text{f}}\right)$$

yields $\Lambda \left(\bar{u}\right)=\bar{x}$ almost everywhere. We have that $\hat{J}$ is sequentially weakly lower semi-continuous, hence


$$\hat{J}\left(\bar{u},\bar{x}\right)\leq \underset{k\to \infty }{\text{lim}}\text{inf}\;\hat{J}\left(u^{k_{\text{l}}},x^{k_{\text{l}}}\right)=\underset{\Omega }{\text{inf}}\;\hat{J}\left(u,x\right),$$

which yields the desired result.∎

**Theorem 4.4.** *Let* $J_{A},J_{S}$ *be given as in (3.2) and (3.3). Then game (AS) has a SE* $\left(x^{*},u_{p}^{*},u_{b}^{*},u_{c}^{*}\right)$ *on* ${\left(H^{1}\left(0,T_{f}\right)\right)}^{4}\times \Omega_{1}\times \Omega_{2}\times R_{S}$ *such that* $x^{*}$ *is a solution of (2.5), and* $\left(u_{p}^{*},u_{b}^{*},u_{c}^{*}\right)$ *minimize* $J_{A}$ *in* $\Omega_{1}\times \Omega_{2}\times R_{S}$ *.*

*Proof.* For proving existence of a minimizer of $J_{S}$ , given in (3.3), we can follow the same arguments in theorem 4.3, owing to the fact that $\Omega_{3}$ is a closed subspace of a Hilbert space and $J_{S}$ is coercive in $\Omega_{3}$ , which yields a convergent subsequence $\left(u_{c}^{m_{l}}\right)$ of a minimizing sequence $\left(u_{c}^{m}\right)$ for $J_{S}$ . The compactness result yields strong convergence of a subsequence $\left(x^{m_{l}}\right)$ in ${\left(H^{1}(0,T_{f})\right)}^{4}$ such that $x^{m_{l}}=\Lambda \left(u_{c}^{m_{l}}\right)$ . Then, we obtain the best-response $R_{C}$ curve of the signalling pathways. Once $R_{C}$ is obtained, we can prove the existence of SE by using the same arguments again. ∎

The Frechét differentiability of $J_{A},J_{S}$ gives rise to the first order necessary optimality conditions as follows: for the minimization problem (3.2), the optimality system is given as


(4.6) $$\begin{array}{l} \frac{dL}{dt}=\gamma L+\frac{1}{2}a_{1}\alpha LC+a_{2}C-a_{3}\alpha u_{p}LT,\;L\left(0\right)=L_{0}\\ \frac{dC}{dt}=b_{1}\left(\alpha -\frac{1}{2}C\right)L-b_{2}\left(\frac{1}{2}H_{0}+\frac{1}{2}C\right)C+b_{3}\left(\frac{1}{2}H_{0}-\frac{1}{2}C\right)-b_{4}\frac{u_{b}}{k+bu_{c}}C,\;C\left(0\right)=C_{0}\\ \frac{dT}{dt}=\omega -d_{1}\left(L+C\right)T+d_{2}u_{p}T,T\left(0\right)=T_{0}\\ \frac{dM}{dt}=-d_{3}M+d_{4}u_{b},\;M\left(0\right)=M_{0}, \end{array}$$

(4.7) $$\begin{array}{l} \frac{d\lambda_{1}}{dt}=-2(\tau -1)[G-(L+C)]-\lambda_{1}(\gamma +\frac{1}{2}a_{1}\alpha C-a_{3}\alpha u_{p}T)-\lambda_{2}[b_{1}(\alpha -\frac{1}{2}C)]+\lambda_{3}d_{1}T,\\ \frac{d\lambda_{2}}{dt}=-2(\tau -1)[G-(L+C)]-\lambda_{1}(\frac{1}{2}a_{1}\alpha L+a_{2})-\lambda_{2}(-\frac{1}{2}b_{1}L-\frac{1}{2}b_{2}H_{0}-b_{2}C-\frac{1}{2}b_{3}-\frac{b_{4}u_{b}}{k+bu_{c}})+\lambda_{3}d_{1}T,\\ \frac{d\lambda_{3}}{dt}=-ru_{p}+\lambda_{1}a_{3}\alpha u_{p}L-\lambda_{3}[-d_{1}(L+C)+d_{2}u_{p}],\\ \frac{\lambda_{4}}{dt}=\lambda_{4}d_{3},\\ \lambda_{1}(T_{f})=0,\;\lambda_{2}(T_{f})=0,\;\lambda_{3}(T_{f})=0,\;\lambda_{4}(T_{f})=0, \end{array}$$

(4.8) $$\begin{array}{rl} \langle 2\tau (Z-u_{b})+2\mu u_{b}+\lambda_{2}\frac{b_{4}C}{k+bu_{c}}-\lambda_{4}d_{4},u-u_{b}\rangle_{L^{2}(0,T)} & \geq 0\\ \langle -rT+2\eta u_{p}+\lambda_{1}a_{3}\alpha LT-\lambda_{3}d_{2}T,u-u_{p}\rangle_{L^{2}(0,T)} & \geq 0, \end{array}$$

for all $\left(u_{p},u_{b}\right)\in \Omega_{1}\times \Omega_{2}$ . For the minimization problem (3.3), the optimality system is given as


(4.9) $$\begin{array}{l} \frac{dL}{dt}=\gamma L+\frac{1}{2}a_{1}\alpha LC+a_{2}C-a_{3}\alpha u_{p}LT,\;L\left(0\right)=L_{0}\\ \frac{dC}{dt}=b_{1}\left(\alpha -\frac{1}{2}C\right)L-b_{2}\left(\frac{1}{2}H_{0}+\frac{1}{2}C\right)C+b_{3}\left(\frac{1}{2}H_{0}-\frac{1}{2}C\right)-b_{4}\frac{u_{b}}{k+bu_{c}}C,\;C\left(0\right)=C_{0}\\ \frac{dT}{dt}=\omega -d_{1}\left(L+C\right)T+d_{2}u_{p}T,\;T\left(0\right)=T_{0}\\ \frac{dM}{dt}=-d_{3}M+d_{4}u_{b},\;M\left(0\right)=M_{0}, \end{array}$$

(4.10) $$\begin{array}{l} \frac{d\xi_{1}}{dt}=\gamma -\xi_{1}\left(\gamma +\frac{1}{2}a_{1}\alpha C-a_{3}\alpha u_{p}T\right)-\xi_{2}\left[b_{1}\left(\alpha -\frac{1}{2}C\right)\right]+\xi_{3}d_{1}T\\ \frac{d\xi_{2}}{dt}=1-\xi_{1}\left(\frac{1}{2}a_{1}\alpha L+a_{2}\right)-\xi_{2}\left(-\frac{1}{2}b_{1}L-\frac{1}{2}b_{2}H_{0}-b_{2}C-\frac{1}{2}b_{3}-\frac{b_{4}u_{b}}{k+bu_{c}}\right)+\xi_{3}d_{1}T\\ \frac{d\xi_{3}}{dt}=\xi_{1}a_{3}\alpha u_{p}-\xi_{3}\left[-d_{1}\left(L+C\right)+d_{2}u_{p}\right]\\ \frac{\xi_{4}}{dt}=\xi_{4}d_{3},\\ \xi_{1}\left(T_{\text{f}}\right)=0,\;\xi_{2}\left(T_{\text{f}}\right)=0,\;\xi \left(T_{\text{f}}\right)=0,\;\xi_{4}\left(T_{\text{f}}\right)=0, \end{array}$$

(4.11) $${\left\langle 2\nu u_{\text{c}}+bu_{\text{b}}\frac{\xi_{2}b_{4}C-1}{{\left(k+bu_{\text{c}}\right)}^{2}}\right\rangle }_{L^{2}\left(0,T\right)}\geq 0,$$

for all $u_{c}\in \Omega_{3}$ . Here, ${\langle \cdot ,\cdot \rangle }_{L^{2}(0,T)}$ is the standard $L^{2}(0,T)$ inner product, which is defined as follows:


$${\left\langle f,g\right\rangle }_{L^{2}\left(0,T\right)}=\int_{0}^{T}f\left(t\right)g\left(t\right)dt.$$

## 5. Numerical schemes for solving Nash and Stackelberg equilibria

In this section, we will present the numerical schemes used to determine the NE and SE of the game (AS). In order to address the nonlinear coupling among the strategies adopted by the players, a relaxation scheme (e.g. [41]) is employed for the NE. The relaxation scheme is implemented in algorithm (5.1).


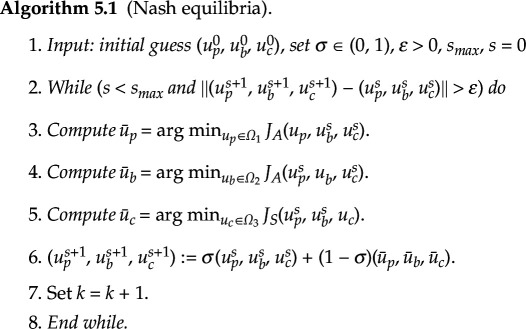


To compute the SE, the following algorithm uses a sequential implementation of the relaxation method. To achieve efficacy, mAbs must possess the ability to anticipate and predict the optimal response of signalling pathways to their initial therapeutic intervention. The resolution of the optimization problem linked to the signalling pathways engenders anticipation. By solving the optimization problem of the mAbs using the optimal responses of signalling pathways as substitutes, the mAbs can determine the most effective strategies to employ. Using optimal doses of Pembrolizumab as strategies for mAbs will stimulate T cells to attack the signalling pathways. The mAbs will have alternative optimal strategies, using optimal Brentuximab doses to deliver chemotherapy in case the optimal Pembrolizumab doses are not effective enough to destroy the signalling pathways entirely [42].


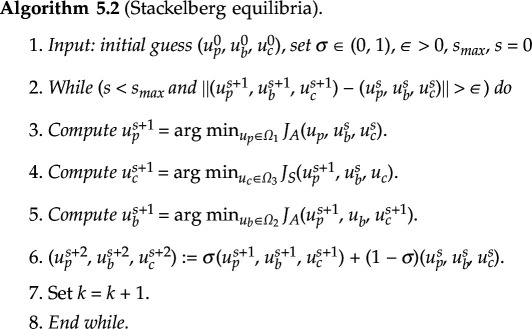


**Remark 5.1.** *The difference between the aforementioned algorithms for computing the Nash and Stackelberg equilibria are that in the case of Nash equilibria, the three strategies* $\left(u_{p},u_{b},u_{c}\right)$ *are updated simultaneously using the previous iterate values, whereas in the case of Stackelberg equilibria,* $u_{p}$ *is updated first, followed by* $u_{c}$ *using the current value of* $u_{p}$ *, followed by* $u_{b}$ *using the current values of* $u_{p},u_{c}$ *. In essence, one can think of the Nash algorithm as a Gauss–Jacobi iterative method while the Stackelberg algorithm as a Gauss–Seidel iterative method.*

In the algorithms mentioned above, it is necessary to select a relaxation factor, denoted as σ , that is sufficiently small to ensure convergence. The convergence of algorithm (5.1) can be proved using analogous arguments in [41]. We provide a sketch of the proof of convergence of algorithm (5.2). For this purpose, we define a map $N:\Omega \to {\left(H^{1}\left(0,\;T_{f}\right)\right)}^{8}$ by N(u)=w . Here, $w={\left(\gamma_{1},\;\gamma_{2},\gamma_{3},\gamma_{4},\;\xi_{1},\;\xi_{2},\xi_{3},\;\xi_{4}\right)}^{T}$ denotes the adjoint variables, which are the solutions to the corresponding adjoint equations (4.7) and (4.10). By demonstrating the boundedness and Lipschitz properties of the adjoint variables using Gronwall’s inequality, similar to lemma 4.2, we can conclude that the map N is both bounded and Lipschitz. For the sake of clarity,


$$\begin{array}{ll} |\xi_{4}\left(t\right)| & =|\xi_{4}\left(T_{\text{f}}\right)+\int_{t}^{T_{\text{f}}}d_{3}\xi_{4}\left(s\right)\;\text{d}s|\\ & \leq |\xi_{4}\left(T_{\text{f}}\right)|+|\int_{t}^{T_{\text{f}}}d_{3}\xi_{4}\left(s\right)\;\text{d}s|\\ & \leq |\xi_{4}\left(T_{\text{f}}\right)|+\int_{t}^{T_{\text{f}}}d_{3}|\xi_{4}\left(s\right)|\text{d}s\\ & \leq |\xi_{4}\left(T_{\text{f}}\right)|\text{exp}\;\left(\int_{t}^{T_{\text{f}}}d_{3}\;\text{d}s\right)\\ & =|\xi_{4}\left(T_{\text{f}}\right)|\text{exp}\;\left(d_{3}\left(T_{\text{f}}-t\right)\right). \end{array}$$

The optimality systems for the minimization problems (3.2) and (3.3) are given to be uniquely solvable, and we now consider $B\left(u_{p}^{*},u_{b}^{*},u_{c}^{*}\right)$ to be the largest closed ball of Ω centred at a SE $\left(u_{p}^{*},u_{b}^{*},u_{c}^{*}\right)$ for the game (AS). We define a map $A:B\left(u_{p}^{*},u_{b}^{*},u_{c}^{*}\right)\to B\left(u_{p}^{*},u_{b}^{*},u_{c}^{*}\right)$ by $A\left(u_{p},u_{b},u_{c}\right)=\sigma \left(u_{p},u_{b},u_{c}\right)+\left(1-\sigma \right)\left(u_{p},u_{b},u_{c}\right)$ , which is well defined owing to the assumption on $B\left(u_{p}^{*},u_{b}^{*},u_{c}^{*}\right)$ . Furthermore, A is a contraction map in $B\left(u_{p}^{*},u_{b}^{*},u_{c}^{*}\right)$ for two reasons: (i) $B\left(u_{p}^{*},u_{b}^{*},u_{c}^{*}\right)\subset L^{2}\left(\left[0,\;T_{f}\right],R\right)$ , which is a complete space, and (ii) its Lipschitz property, which is achieved by both x and w being Lipschitz. As a result, the map A has a unique fixed point. This shows that the algorithm (5.2) is convergent in $B\left(u_{p}^{*},u_{b}^{*},u_{c}^{*}\right)$ to a SE.

Furthermore, in each iteration step of the aforementioned algorithms, the minimization problem for each player in the game (3.4) must be solved, which involves solving the optimality systems (4.6)–(4.8) and (4.9)–(4.11). We use the traditional forward Euler method for solving the ODE systems (4.6) and (4.9) and their respective adjoints (4.7) and (4.10). For solving the optimality conditions (4.8) and (4.11), we use a projected nonlinear conjugate gradient (NCG) scheme. It belongs to the category of nonlinear optimization schemes, wherein the objective functional exhibits differentiability in relation to the optimization variables. The reduced functional corresponding to either of the minimization problems is denoted generically by ${\hat{J}}_{o}$ , and the associated optimization variable as u . Starting with the initial guess $u_{0}$ , we compute the first descent direction as


$$d_{0}=g_{0}:=\nabla_{u}{\hat{J}}_{o}\left(u_{0}\right),$$

where $\nabla {\hat{J}}_{o}$ is given by (4.8) or (4.11). The search directions are then obtained recursively as


(5.1) $$d_{k+1}=-g_{k+1}+\beta_{k}d_{k},$$

where $g_{k}=\nabla \hat{J}\left(u_{k}\right),\;k=0,1,\ldots$ and the parameter $\beta_{k}$ is chosen according to the formula of Hager–Zhang [43] given by


(5.2) $$\beta_{k}^{HG}=\frac{1}{d_{k}^{T}y_{k}}{\left(y_{k}-2d_{k}\frac{\|y_{k}{\|}_{l^{2}}^{2}}{d_{k}^{T}y_{k}}\right)}^{T}g_{k+1},$$

where $y_{k}=g_{k+1}-g_{k}$ . Next, a conjugate gradient descent step is used to compute the new optimization variable iterate


(5.3) $$u_{k+1}=u_{k}+\alpha_{k}\;d_{k},$$

where k is an index of the iteration step and $\alpha_{k}>0$ is a step length obtained using a line search algorithm. For this line search, we use the following Armijo condition of sufficient decrease of $\hat{J}$


(5.4) $${\hat{J}}_{o}\left(u_{k}+\alpha_{k}d_{k}\right)\leq {\hat{J}}_{o}\left(u_{k}\right)+\delta \alpha_{k}{\left\langle \nabla_{u}{\hat{J}}_{o}\left(u_{k}\right),d_{k}\right\rangle }_{L^{2}},$$

where 0<δ<1/2 and the scalar product ${\langle u,v\rangle }_{L^{2}}$ represents the standard $L^{2}([0,T])$ inner product for the minimization problems (3.2) and (3.3). The gradient update step is finally combined with the following projection step to ensure that the iterates stay in the admissible sets.


(5.5) $$u_{k+1}=P_{U}\left[u_{k}+\alpha_{k}\;d_{k}\right],$$

where


$$P_{U}\left[u\right]=\left(\text{max}\left\{0,\left\{N_{i},u_{i}\right\}\right\},\forall i=1,\ldots ,s\right)$$

with $U=\Omega_{1},\Omega_{2}$ or $\Omega_{3}$ , and $N_{i}=Z_{i},G_{i}$ or $r_{i}$ , corresponding to the minimization problems in the above algorithms,. The projected NCG scheme can be summarized in the following algorithm:


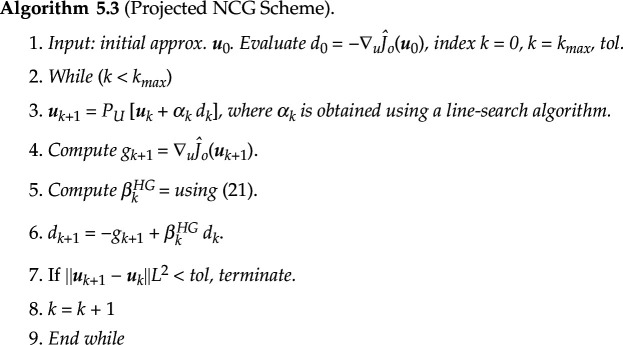


## 6. Numerical results

We present the numerical results of the NE and SE for the differential game (AS). For this purpose, we choose our non-dimensionalized scaling parameters as $q_{1}=10^{-2},\;q_{2}=10^{-1},\;q_{3}=10^{-6},\;q_{4}=1,\;q_{5}=0.5,\;q_{6}=q_{8}=1$ and $q_{7}=4$ . With the original time interval as [0,200] days, this transformation yields the final time $T_{f}=100$ . In the following two cases, the parameter values in the functionals $J_{A},\;J_{S}$ are given as τ=1.8328, Z=1.425, G=0.98039, r=0.01 . We choose the values of the weights in the functional $J_{A}$ as μ=1,η=0.5 , and the weight in the functional $J_{S}$ as ν=0.5 .

**Figure 2.**
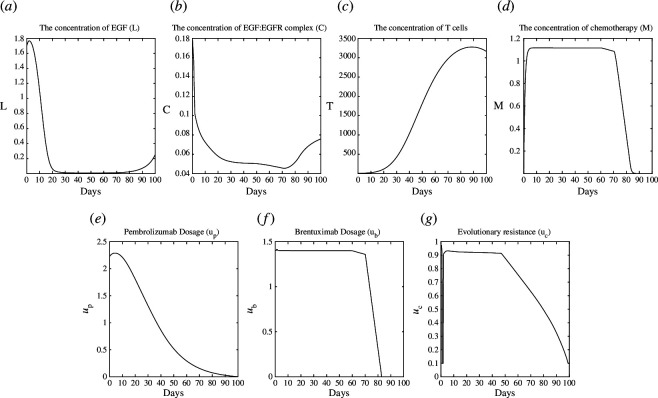
Test case 1: plot of NE. (*a*): L, (*b*): C, (*c*):T, (*d*): M, (*e*):u_p_, (*f*): u_b_ (*g*): u_c_.

**Figure 3.**
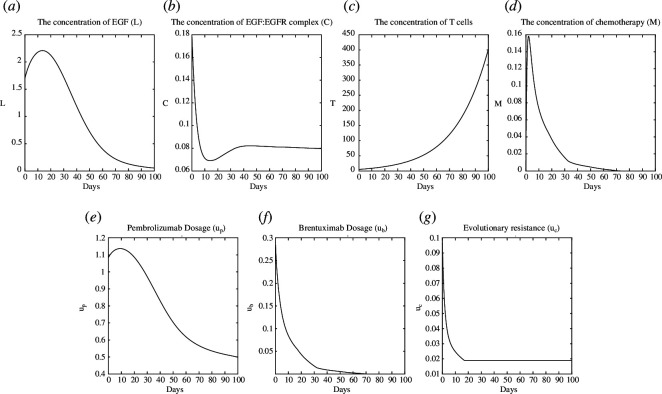
Test case 1: plot of SE. (*a*): L, (*b*): C, (*c*):T, (*d*): M, (*e*):u_p_, (*f*): u_b_ (*g*): u_c_.

For the test case 1 , the patient data are generated as follows: we first simulate the following reduced ODE system for


(6.1) $$\begin{array}{l} \frac{dL}{dt}=\gamma L+\frac{1}{2}a_{1}\alpha LC+a_{2}C-a_{3}\alpha LT,\;\;L\left(0\right)=L_{0}\\ \frac{dC}{dt}=b_{1}\left(\alpha -\frac{1}{2}C\right)L-b_{2}\left(\frac{1}{2}H_{0}+\frac{1}{2}C\right)C+b_{3}\left(\frac{1}{2}H_{0}-\frac{1}{2}C\right)-b_{4}C,\;\;C\left(0\right)=C_{0}\\ \frac{dT}{dt}=\omega -d_{1}\left(L+C\right)T+d_{2}T,\;\;T\left(0\right)=T_{0}\\ \frac{dM}{dt}=-d_{3}M+d_{4},\;\;M\left(0\right)=M_{0}, \end{array}$$

with the non-dimensionalized parameters values γ=0.02925, $\;a_{1}=0.09941,\;a_{2}=0.43985,$ $\;a_{3}=0.06629,$ $\;b_{1}=0.06591,\;b_{2}=0.21051,\;b_{3}=0.13991,\;b_{4}=0.17899,$ $\;\omega =0.06721,\;d_{1}=0.02272,$ $\;d_{2}=0.08395,$ $\;d_{3}=1,$ $d_{4}=0.7970$ and initial conditions C(0)=1.7, L(0)=0.18, T(0)=5, M(0)=0 . The reduced model (6.1) is obtained by excluding treatments $u_{p},\;u_{b}$ , and the evolutionary resistance $u_{c}$ from (2.1). We have the term $u_{b}/(k+bu_{c})$ in (2.1), where the parameters k, b are given as k=0.1, b=5 (see [34]). We also provide initial conditions for the remaining variables as $H_{0}=0.1,\;R_{0}=0.105,\alpha =0.055$ . The non-dimensionalized initial guess for NE is given as $\left(u_{p}^{0},u_{b}^{0},u_{c}^{0})=(0,0,0\right)$ . The assumption is motivated by the fact that all of the players are actively engaged in the game at the same time. Conversely, within the context of the Stackelberg scenario, player A assumes the role of the leader and initiates the first action by administering Pembrolizumab, subsequently prompting player S to respond. If this strategy fails to effectively manage the developing resistance $u_{c}$ , an alternative approach involving the administration of Brentuximab will be implemented by A . Based on this, we choose $\left(u_{p}^{0},u_{b}^{0},u_{c}^{0})=(0.5,0,0\right)$ as our non-dimensionalized first guess for SE. We first solve the game (3.4) for NE and SE using (5.1) and (5.2), respectively. The illustrating plots of the NE and SE are shown in figures 2 and 3, respectively.

Pembrolizumab and Brentuximab are administered at the MTD, and the use of Brentuximab is repeated many times at the same amount as shown in figure 2. We also observe that $u_{b}$ transports chemotherapy at high doses and the same amount multiple times. With treatment, L and C go down, but the players play the game simultaneously, and the mAbs cannot predict how the signalling pathways will act, so they cannot adjust their treatment strategies appropriately. This leads to the Nash game, in which the mAbs give up control of the game to the signalling pathways. Therefore, L and C continue to spread again because it is a consequence of the successful adaptations that the signalling pathways have made by evolving their resistance.

As shown in figure 3, the regulation or control of L and C is achieved through the responses of the monoclonal antibodies to the evolutionary resistance of signalling pathways. There is an imbalance in this game that makes it impossible for the signalling pathways to predict or adapt to treatments that the mAbs have not yet given. This results in the emergence of SE, where the monoclonal antibodies take on a leadership role. The monoclonal antibodies track the strategies of the signalling pathway by having $u_{b}$ mimic the behaviour of $u_{c}$ , decreasing $u_{p}$ dosage and maintaining it at a lower level if $u_{c}$ increases again. After t=10 , $u_{c}$ exhibited a consistent or unvarying pattern, resulting in a chronic L and C .

**Figure 4.**
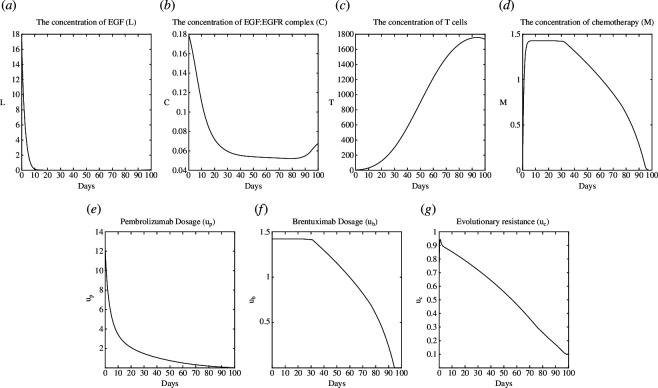
Test case 2: plot of NE. (*a*): L, (*b*): C, (*c*):T, (*d*): M, (*e*):u_p_, (*f*): u_b_ (*g*): u_c_.

**Figure 5.**
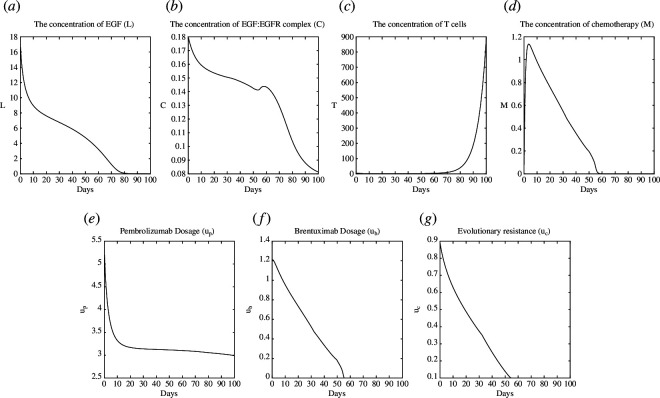
Test case 2: plot of SE. (*a*): L, (*b*): C, (*c*):T, (*d*): M, (*e*):u_p_, (*f*): u_b_ (*g*): u_c_.

In the test case 2, we simulate the ODE system with the non-dimensionalized parameter values $\gamma =0.000815,\;a_{1}=0.11022,$ $\;a_{2}=0.45410,\;a_{3}=0.07240,$ $\;b_{1}=0.06599,\;b_{2}=0.17558,$ $\;b_{3}=0.01633,\;b_{4}=0.1006228,$ $\;\omega =0.07232,\;d_{1}=0.03173,\;d_{2}=0.05256,\;d_{3}=1,$ $\;d_{4}=1.0052,\;k=0.1,\;b=5$ and initial conditions $C_{0}=17,\;L(0)=0.18,\;T(0)=5,\;M(0)=0$ . The initial conditions for the remaining variables are $H_{0}=0.8,\;R_{0}=0.5,\;\alpha =0.1$ . Given that γ denotes the growth rate of monovalent ligands (EGF) and ω denotes the rate of circulating T cells, the value of γ has decreased, whereas the value of ω has increased. The chosen parameter values indicate that the patient in the test case 2 has a strengthened immune system compared with the patient in the test case 1. The non-dimensionalized initial guesses for NE and SE are given as $\left(u_{p}^{0},u_{b}^{0},u_{c}^{0})=(0,0,0\right)$ , and $\left(u_{p}^{0},u_{b}^{0},u_{c}^{0})=(3,0,0\right)$ , respectively. The illustrating plots of the NE and SE are shown in figures 4 and 5, respectively.

In figure 4, It can be seen that L has strictly decreased as a result of the reason that this test case represents a patient with a strengthened immune system compared with the previous test case. Because of the same reason, the chemotherapy dosage and its transporter $u_{b}$ are lower in this case compared with the test case 1. However, the resurgence of C has been aided by the failure to eliminate evolutionary resistance after t=90 owing to the lack of utilization of asymmetry in the game.

The signalling pathways L and C have decreased owing to the monoclonal antibody’s response to their evolutionary resistance; however, the mAbs will continue to apply the most effective treatment strategies to eliminate any remaining resistance and stop its growth once more, as we see in figure 5. $u_{p}$ activates T cells at lower levels compared with the test case 1 because this test case represents a patient with a strengthened immune system compared with the previous test case. We observe that $u_{b}$ exhibits a comparable pattern of low concentration based on the level of cancer resistance $u_{c}$ at different time points.

The results of the two cases suggest that dynamic therapy designs that explicitly account for the evolutionary dynamics of resistance could replace the current treatment protocols that apply the drugs at MTD to take advantage of signalling pathway asymmetries. Our computational findings imply that the therapy of signalling pathways is analogous to a Stackelberg game, where monoclonal antibodies influence resistance evolution and total signalling pathway load. Therefore, compared with Nash equilibrium, Stackelberg equilibrium yields superior outcomes.

## 7. Conclusions

In this paper, we proposed a new framework in which a non-cooperative evolutionary differential game is formulated between mAbs and signalling pathways in OC. For this purpose, we employed a novel evolutionary mathematical model to simulate the dynamics of signalling pathways, incorporating the phenomenon of resistance evolution. We then solved a differential game to obtain the NE and SE. The relaxation scheme and a sequential version of the relaxation scheme were used to compute NE and SE, respectively. Based on our numerical experiments, the mAbs should prioritize SE over NE in order to improve OC patient outcomes.

## Data Availability

The parameter values and data used to generate the results are already present in the paper. The corresponding codes to replicate the results are available via Dryad [44].
